# Reply to ‘Comment on “Tumour-agnostic drugs in paediatric cancers”’

**DOI:** 10.1038/s41416-020-01104-z

**Published:** 2020-10-01

**Authors:** Julia C. Chisholm, Fernando Carceller, Lynley V. Marshall

**Affiliations:** Paediatric and Adolescent Oncology Drug Development Team, Children and Young People’s Unit, Royal Marsden Hospital and Institute of Cancer Research, Sutton, UK

**Keywords:** Paediatric cancer, Sarcoma, Molecular medicine

Following our Comment on Tumour-agnostic drugs in paediatric cancers (*Br. J. Cancer* 122:1425–1427, 2020), the National Institute for Health and Care Excellence completed its consultation process on larotrectinib. With an improved pricing agreement, it was recommended in April 2020 for use within the Cancer Drugs Fund (CDF) for neurotrophic tyrosine receptor kinase (NTRK) fusion-positive solid tumours in adults and children with locally advanced or metastatic disease or where surgery could cause severe health problems and no satisfactory treatment options exist. It was recognised during the consultation that patients and clinicians may wish to access the drug earlier in treatment and that further information is required to determine optimal positioning within the patient pathway (https://www.nice.org.uk/guidance/ta630/documents/final-appraisal-determination-document). The conditions for use associated with the CDF will allow further real-world data collection to inform future marketing authorisation.

In this issue, Oliver et al. report an interesting case of a child with infantile fibrosarcoma (IFS) with a canonical ETV6-NTRK3 fusion who failed conventional chemotherapy, developed rapid resistance to larotrectinib and subsequently showed resistance to the second-generation TRK inhibitor selectrectinib (Loxo-195). They elegantly map the molecular evolution of the resistance. They conclude that comparative clinical trials beyond basket studies are needed to learn when and how to deploy drugs like larotrectinib and that until such evidence is available, cautious use of TRK inhibitors in children should be advocated.

In our view, TRK inhibitors should only be used under direct supervision by oncologists experienced in the use of such targeted agents. While agreeing that the place of larotrectinib in treatment of tumours with NTRK mutations remains to be defined, the unusually aggressive and ultimately fatal case with incompletely resectable disease Oliver et al. present highlights the potential role for tumour-agnostic drugs like larotrectinib to facilitate surgery or prolong life, emphasises the need to address emergent resistance mechanisms and compels us to urgently explore whether earlier use of these agents may lead to better outcomes for patients.

The level of evidence required to introduce tumour-agnostic agents such as larotrectinib into frontline treatment requires careful consideration. The tension is to ensure equipoise in trying to design a randomised/comparative registration level trial for a heterogeneous group of tumours with a known but rare target, when a new class of drugs with high efficacy, favourable short-term toxicity profile and an oral formulation suitable for the youngest of infants becomes available. This may be even more important for patients likely to survive their disease, where long-term chemotherapy side effects or morbid surgical approaches may impact significantly on quality of life.

IFS, a tumour of young children and the most common paediatric malignancy harbouring an NTRK fusion, might present a more uniform population for comparative or randomised paediatric trials of TRK inhibitors. In the experience of the European paediatric Soft tissue Sarcoma Group, only 50 cases of non-metastatic IFS were registered in 14 countries in 7 years,^1^ of which 19 (38%) were resected as primary treatment and 31 (62%) were Group III (macroscopic residual disease/biopsy only). Overall, 30/31 (97%) Group III patients received chemotherapy (mainly vincristine and actinomycin D, but including 5 ifosfamide, 2 cyclophosphamide and 1 doxorubicin), 2/31 patients (6%) had mutilating surgery (amputations), 1/31 (3%) was irradiated and 3/31 (10%) died (1 toxicity, 2 disease progression). This study demonstrates that the potential population for performing prospective trials in newly diagnosed IFS is extremely small. Moreover, fatal toxicity, potential late effects of chemotherapy, such as nephrotoxicity, reduced fertility or cardiotoxicity and the need for mutilating surgery are a real risk for these patients.

The ongoing phase II study in newly diagnosed NTRK fusion-positive IFS, solid and brain tumours and relapsed leukaemia in patients <30 years (NCT03834961) is addressing objective response rate to first-line treatment with larotrectinib, with secondary endpoints of toxicity, event-free survival and overall survival and, for IFS, duration of response. It will provide further information on the use of the drug in frontline and results are eagerly awaited. Meanwhile, the role of the second-generation TRK inhibitor seletrectinib is also under investigation in a phase I/II study (NCT03215511) and an expanded access programme (NCT03206931). Additional TRK inhibitors such as entrectinib (Roche) and repotrectinib (Turning Point Therapeutics) are in development. While acknowledging the excellent points raised by Dr. Oliver and colleagues, it remains our view that given the favourable toxicity profile compared to conventional options and the demonstrated high response rates, TRK inhibitors constitute a highly attractive frontline option in IFS that for many patients may allow definitive, curative surgery before development of drug resistance.

## Data Availability

Not applicable.
